# Loss of *ZNF32* augments the regeneration of nervous lateral line system through negative regulation of *SOX2* transcription

**DOI:** 10.18632/oncotarget.11895

**Published:** 2016-09-08

**Authors:** Yuyan Wei, Kai Li, Shaohua Yao, Junping Gao, Jun Li, Yanna Shang, Jie Zhang, Le Zhang, Yanyan Li, Xianming Mo, Wentong Meng, Rong Xiang, Jiankun Hu, Ping Lin, Yuquan Wei

**Affiliations:** ^1^ Division of Experimental Oncology, State Key Laboratory of Biotherapy, West China Hospital, Sichuan University, and Collaborative Innovation Center for Biotherapy, Chengdu, P.R. China; ^2^ Division of Cancer Biotherapy, State Key Laboratory of Biotherapy, West China Hospital, Sichuan University, and Collaborative Innovation Center for Biotherapy, Chengdu, P.R. China; ^3^ Laboratory of Stem Cell Biology, State Key Laboratory of Biotherapy, West China Hospital, Sichuan University, and Collaborative Innovation Center for Biotherapy, Chengdu, P.R. China; ^4^ Department of Gastrointestinal Surgery and Laboratory of Gastric Cancer, State Key Laboratory of Biotherapy, West China Hospital, Sichuan University, and Collaborative Innovation Center for Biotherapy, Chengdu, P.R. China; ^5^ Department of clinical medicine, School of Medicine, Nankai University and Collaborative Innovation Center for Biotherapy, Tianjin, P.R. China

**Keywords:** ZNF32, SOX2, regeneration, DNA binding site, NLS

## Abstract

Human zinc finger protein 32 (ZNF32) is a Cys2-His2 zinc-finger transcription factor that plays an important role in cell fate, yet much of its function remains unknown. Here, we reveal that the zebrafish *ZNF32* homologue *zfZNF32* is expressed in the nervous system, particularly in the lateral line system. *ZfZNF32* knock-out zebrafish (*zfZNF^−/−^*) were generated using the CRISPR-associated protein 9 system. We found that the regenerative capacity of the lateral line system was increased in *zfZNF^−/−^* upon hair cell damage compared with the wild type. Moreover, *SOX2* was essential for the *zfZNF32*-dependent modulation of lateral line system regeneration. Mechanistic studies showed that ZNF32 suppressed *SOX2* transcription by directly binding to a consensus sequence (5′-gcattt-32) in the *SOX2* promoter. In addition, ZNF32 localizes to the nucleus, and we have identified that amino acids 1-169 (Aa 1-169) and each of three independent nuclear localization signals (NLSs) in ZNF32 are indispensable for ZNF32 nuclear trafficking. Mutating the NLSs disrupted the inhibitory effect of ZNF32 in *SOX2* expression, highlighting the critical role of the NLSs in ZNF32 function. Our findings reveal a pivotal role for *ZNF32* function in *SOX2* expression and regeneration regulation.

## INTRODUCTION

Transcription factors (TFs) play pivotal roles in a wide range of human biological functions and regulate gene expression by binding to specific cis- regulatory sequences in the promoters of their target genes [1]. The DNA-binding specificities of TFs are a key component of gene regulatory processes. In contrast to the genetic code, the transcriptional regulatory code is far from being deciphered and is determined by the sequence specificity of TFs, by combinatorial cooperation between TFs and by chromatin competence [2]. Identifying the specific DNA sequences bound by TFs contributes to the computational prediction of cis-regulatory modules (CRMs) that regulate gene expression and to the further understanding of TF functions. Cyclic Amplification and Selection of Targets (CASTing), which selects DNA sequences bound by specific proteins, has been used as a rapid and general *in vitro* approach to screen for the DNA-binding sequence of TFs [3].

Cys2-His2 (C2H2) zinc-finger proteins represent the largest class of putative human TFs, which are involved in cellular processes such as proliferation, differentiation, and development [4, 5] and are even associated with many diseases such as cancer [6]. Bioinformatics databases indicate that ZNF32, which belongs to the Krüpple-like family of TF, contains six consecutive typical C2H2 zinc-finger motifs and one degenerate C2H2 zinc-finger motif and may bind DNA for transcriptional regulation. Previously, we showed that *Zfp637*, the mouse homologue of human *ZNF32*, repressed myogenic cellular differentiation [7] and protected cells from oxidative stress-induced premature senescence [8]. All of these findings suggest that *ZNF32* plays an important but overlooked role in cellular processes. Our primary focus in the present study is to investigate the function and mechanism of *ZNF32* in lateral line system regeneration.

Regeneration is an important process of renewal, restoration and growth that responds to natural fluctuations or events that cause disturbance or damage. Every species is capable of regeneration, from bacteria to humans. Zebrafish (*Danio Rerio*) is a powerful vertebrate model to elucidate organ/tissue development [9], regeneration [10] and human disease [11]. At its most basic level, regeneration concerns the molecular processes of gene regulation during cellular proliferation and differentiation. Stem cells hold immense promise for beneficial tissue and organ regeneration. The neural stem cell marker *SOX2* is a key transcription factor that is expressed in pluripotent embryonic stem cells and that is widely expressed in early neuroectoderm and neural progenitor cells during development [12]. *SOX2* levels are tightly regulated, and the dysregulation of *SOX2* expression can cause significant changes in differentiation behavior [13]. *SOX2* is crucial for the development of hair cells in the zebrafish lateral line system and is expressed in numerous groups of cells aside from differentiated hair cells. These observations suggest that *SOX2* is likely to play a major role in the maintenance of precursor cells for hair cell regeneration [14].

In addition, the role of zinc-finger proteins in gene regulation requires their transport from the cytoplasm to the nucleus in all eukaryotic cells. Generally, ions and soluble small molecules can be transported across the nuclear envelope by diffusion, whereas the transport of most nuclear proteins from the cytoplasm is mediated by the nuclear pore complex [15]. Transport is an energy-consuming process [16] and is typically coupled to either cytoplasmic anchoring or the activation of a nuclear localization signal (NLS) by unmasking or modification in TFs [17]. Furthermore, NLS mutants cause protein mislocalization, leading to the disruption of TF function, the alteration of cellular physiological processes and many human diseases such as amyotrophic lateral sclerosis [18] and fragile X syndrome [19]. ZNF32 is a confirmed nuclear protein that functions as a TF, as confirmed in our previous study [20], however, its specific target DNA sequence and NLS have not been reported.

In the present study, we measured the expression profile of the zebrafish ZNF32 homologue *zfZNF32* during zebrafish development. *ZfZNF^−/−^* were generated using the CRISPR/Cas9 system and were used to study the hair cell damage and regeneration in the lateral line system. The target DNA sequences and NLSs of ZNF32 were identified. Moreover, the transcriptional regulation of *SOX2* expression by *ZNF32* was found to be critical for regeneration. Our study unravels a novel and important mechanism for the regulation of *SOX2* and highlights the significance of *ZNF32* in regeneration.

## RESULTS

### *ZfZNF32* expression pattern during early embryonic development

**Figure 1:**
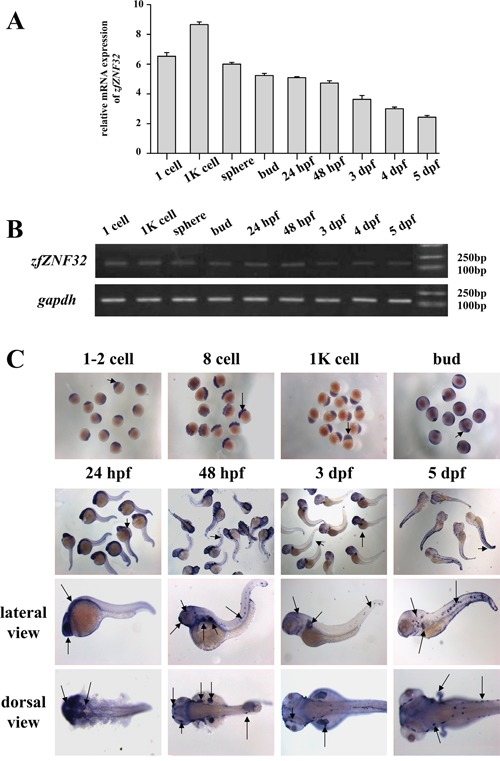
*ZfZNF32* expression pattern during early embryonic development **A.** *ZfZNF32* mRNA was distributed uniformly from the zygote stage to the larval stage according to Q-PCR. **B.** Agarose gel electrophoresis of the (A) PCR product. **C.** Whole-mount *in situ* hybridization revealed the dynamic expression of *zfZNF32* during zebrafish development (from the 1 cell stage to 5 dpf), and *zfZNF32* was mainly restricted to the nervous system and pectoral fin (lateral view and dorsal view), as indicated by arrows.

To explore the role of *ZNF32* in zebrafish, we first evaluated the expression profile of *zfZNF32* during zebrafish development. *ZfZNF32* mRNA was expressed uniformly from the zygote stage to the larval stage and peaked at the 1K cell stage (Figure 1A and 1B). Interestingly, *zfZNF32* was mainly located in the nerve cord at the bud stage. We found that *zfZNF32* was expressed in the neural tube and pectoral fin bud at 24 hour post-fertilization (hpf) and was expressed in the lateral line system and pectoral fin up to 3-5 day post-fertilization (dpf) (Figure 1C). These results show that *zfZNF32* was persistently expressed during early embryonic development but gradually became limited to the nervous system.

### *ZfZNF32* knock-out promotes the regeneration of the lateral line system

**Figure 2:**
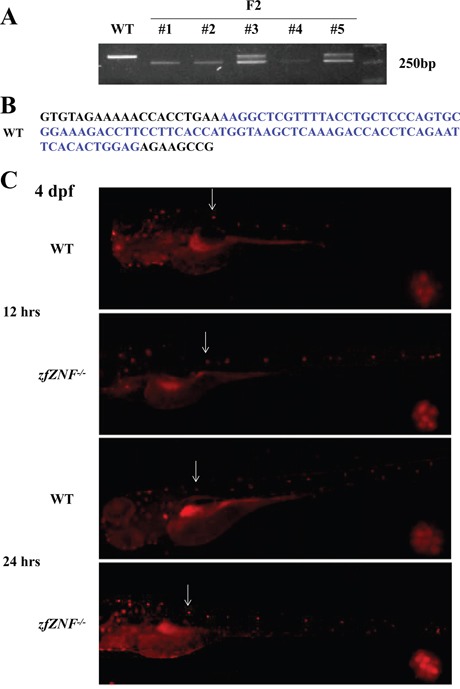
Establishment of *zfZNF32* knock-out zebrafish and lateral line system regeneration **A.** Agarose gel electrophoresis showing the Cas9-mediated *zfZNF^−/−^* with bases deleted compared with WT. **B.** A total of 85 bp were deleted (in blue font) in the *zfZNF^−/−^*. **C.** The regenerative capacity of the lateral line system was increased in 4 dpf *zfZNF^−/−^* at 12 and 24 hrs after neomycin treatment. Hair cells in the lateral line were stained with DASPEI (red fluorescence). The L1 was chosen as the representative regenerating neuromast, as indicated by arrows, and shown in the lower right corner of each figure with 200×magnification.

In order to study the function of *zfZNF*32, Cas9 technology was used to establish permanent *zfZNF^−/−^*. Mutagenesis in F0 zebrafish embryos and the selection of F1 zebrafish were performed as described previously [21]. The *zfZNF^−/−^* selected from F2 were verified by PCR (Figure 2A). Finally, we selected an 85-bp deletion mutation in *zfZNF^−/−^* compared with the wide type (WT) (Figure 2B).

Hair cell regeneration is important for lateral line system repair after injury in non-mammalian vertebrates. The lateral line system comprises two major branches, an anterior part that extends on the head, and a posterior part that extends on the trunk and tail. In this study, The regeneration of the posterior Lateral Line (pLL) has been studied and the L1 was chose as a representative neuromast. Considering that the *zfZNF32* was mainly expressed in the lateral line system, we used neomycin, which induces mature hair cell death, to explore the role of *zfZNF32* in the regeneration of the lateral line system. WT and *zfZNF^−/−^* at 4 dpf were treated with 400 μM neomycin for 1 hr. DASPEI assays showed that the regenerative capacity of the lateral line system was increased at 12 and 24 hrs after hair cell injury in *zfZNF^−/−^* compared with WT (Figure 2C). These results suggest that *zfZNF32* down-regulation promoted the regenerative capacity of the zebrafish lateral line system.

### ZNF32 negatively regulates *SOX2* expression

**Figure 3:**
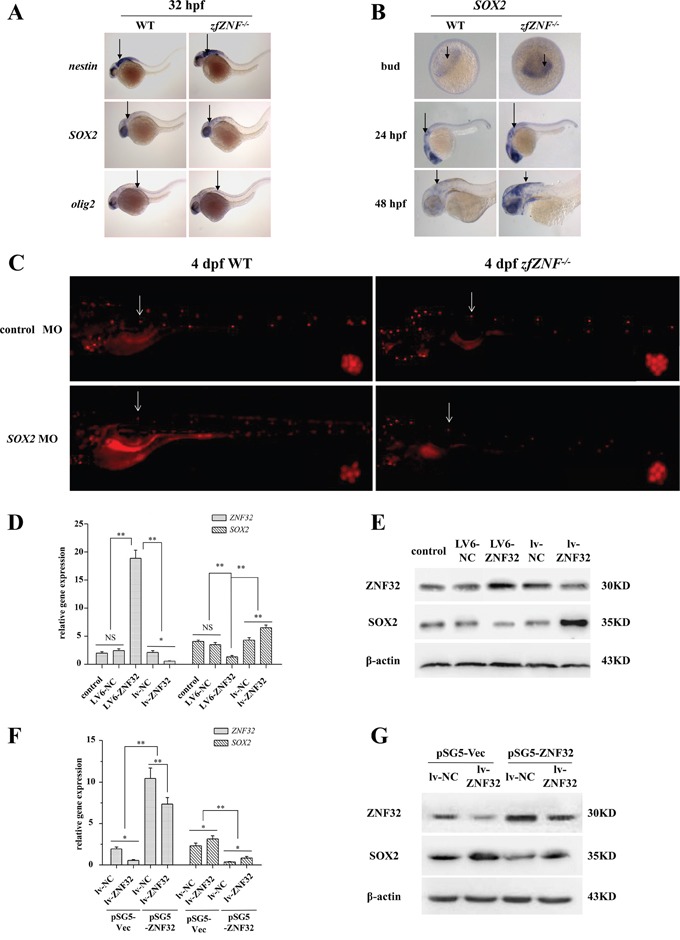
ZNF32 negatively regulates *SOX2* expression *in vivo* and *in vitro* **A.** The early nerve stem cell factors *nestin*, *SOX2* and *olig2* were detected by WISH at 32 hpf in *zfZNF^−/−^* and WT. mRNA expression is indicated by arrows. **B.** WISH revealed *SOX2* expression in *zfZNF^−/−^* and WT at bud, 24 hpf and 48 hpf stages. *SOX2* mRNA expression is indicated by arrows. **C.** The regenerative capacity of the lateral line system was decreased in 4 dpf *zfZNF^−/−^* microinjected with *SOX2* MO compared with control MO at 24 hrs after neomycin treatment. Hair cells in the lateral line were stained with DASPEI (red fluorescence). The L1 was chosen as the representative regenerating neuromast, as indicated by arrows, and shown in the lower right corner of each figure with 200×magnification. Relative gene expression of *ZNF32* and *SOX2* in BE(2)-C cell lines was detected at the mRNA level by Q-PCR **D.** and at the protein level by western blotting **E.** Untreated BE(2)-C cells served as controls. BE(2)-C lv-NC and BE(2)-C lv-ZNF32 cells were transiently transfected with pSG5-Vec and pSG5-ZNF32. Q-PCR and western blotting were performed to detect the relative expression of *ZNF32* and *SOX2* at the mRNA **F.** and protein level **G.** All of the quantitative values are presented as the means±S.D. *P< 0.05, **P< 0.01. NS, not significant.

Nerve stem cell factors play crucial roles in nerve regeneration [22]. To investigate the mechanism of *zfZNF32* in lateral line system regeneration, we examined the expression of the early nerve stem cell factors *nestin*, *SOX2* and *olig2* by WISH. *SOX2* expression, but not *nestin* and *olig2*, was increased at 32 hpf in *zfZNF^−/−^* compared with the WT (Figure 3A). Moreover, elevated *SOX2* expression was observed in *zfZNF^−/−^* from the bud stage to 48 hpf (Figure 3B and Supplementary Figure S1A). To further explore whether *SOX2* is involved in the *zfZNF32*-regulated regeneration of the lateral line system, *zfZNF^−/−^* embryos were microinjected with *SOX2* morpholino (MO) or control MO at the one-cell stage and then treated with 400 μM neomycin for 1 hr at 4 dpf. Decreased *SOX2* expression was observed in *zfZNF^−/−^* and WT microinjected with *SOX2* MO at 4 dpf by Q-PCR (Supplementary Figure S1B). DASPEI assays showed that knocking down *SOX2* reduced the regenerative capacity of the lateral line system in *zfZNF^−/−^* at 24 hrs after hair cell injury (Figure 3C). Together, our data indicate that *SOX2* is essential for the *zfZNF32*-dependent modulation of lateral line system regeneration in zebrafish.

To determine whether *ZNF32* negatively regulated *SOX2* expression in a human cell line, stable ZNF32 over-expression (termed LV6-ZNF32) and stable shRNA-mediated ZNF32 knockdown (termed lv-ZNF32) were established in BE(2)-C cells. Q-PCR and western blotting showed that *SOX2* expression was increased by *ZNF32* knockdown and decreased by *ZNF32* over-expression (Figure 3D and 3E). Moreover, transient transfection of pSG5-ZNF32 into BE(2)-C lv-NC and BE(2)-C lv-ZNF32 to rescue *ZNF32* expression revealed that the elevated expression of *SOX2* was counterbalanced by the re-expression of *ZNF32* in BE(2)-C lv-ZNF32 cells (Figure 3F and 3G). Taken together, these results suggest that *SOX2* expression is negatively regulated by *ZNF32* both in zebrafish and in human BE(2)-C cells.

### ZNF32 directly binds to the *SOX2* promoter to suppress *SOX2* transcription

**Figure 4:**
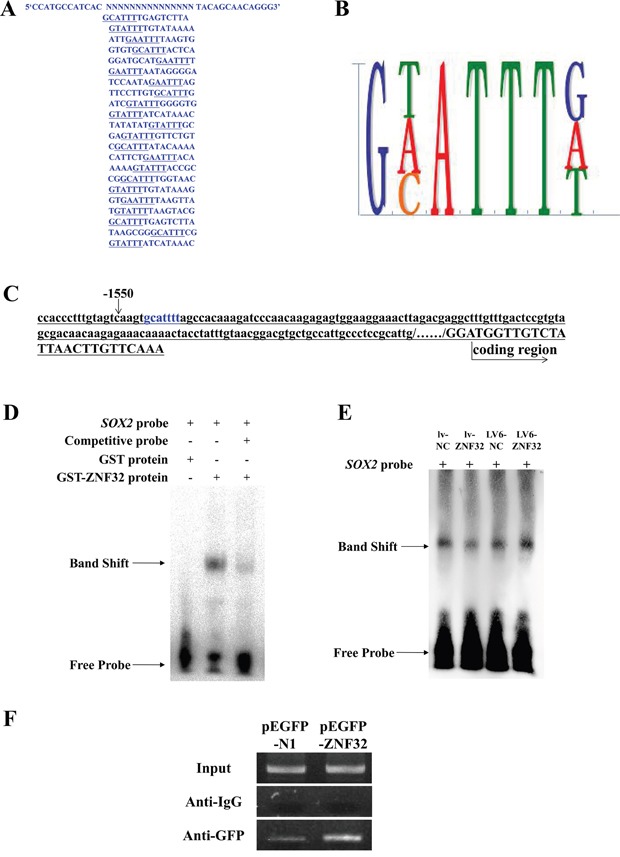
ZNF32 directly binds the *SOX2* promoter to suppress *SOX2* transcription **A.** The DNA binding sites were identified by CASTing. The DNA sequences of the 22 clones are shown. **B.** Analyses of the sequences of the 22 clones revealed the ZNF32-DNA binding site as 5′- g(a/c/t)attt -32. **C.** The 2-kb region upstream of the human *SOX2* promoter was from the NCBI web server. Blue characters mark the DNA binding site, 5′- gcattt -32, located at -1545. **D.** EMSA revealed that the *SOX2* probe was preferentially bound by the ZNF32 protein compared with GST, and the binding capacity was weakened as competitive probe was added *in vitro*. The band shift revealed the protein-DNA binding capacity. **E.** EMSA showing that protein-probe binding corresponded to nuclear ZNF32 protein expression. Nuclear proteins were isolated from the BE(2)-C stable cell lines lv-NC (lane 1), lv-ZNF32 (lane 2), LV6-NC (lane 3), LV6-ZNF32 (lane 4). The band shift changed with the ZNF32 expression level. **F.** GFP-ChIP PCR results indicated that ZNF32 over-expression increased the protein-DNA binding ability, suggesting that ZNF32 specifically binds the *SOX2* promoter sequence. IgG was used as a negative control.

Based on the zinc-finger domains and the localization of ZNF32, we inferred that ZNF32 may act as a TF. However, the specific DNA binding sequence of ZNF32 is not known. We used CASTing to identify the consensus DNA binding sites of ZNF32. The GST-ZNF32 fusion protein was expressed in *E. coli* BL21(DE3) star competent cells and detected by SDS-PAGE analysis (Supplementary Figure S2A). The soluble GST-ZNF32 fusion protein was identified by SDS-PAGE and western blotting following purification and digestion by Factor Xa (Supplementary Figures S2B and S2C). A 55-base PCR product containing a random set of 15 bases was mixed with bead-GST-ZNF32 complexes to form ZNF32-DNA complexes, which forms the first CASTing cycle. After 10 CASTing cycles, the DNA binding site was finally obtained by sequencing 22 DNA clones (Figure 4A) and identified as 5′-g(a/c/t)attt-32 (Figure 4B).

As described above, *SOX2* expression was negatively regulated by ZNF32. To investigate whether ZNF32 directly regulates *SOX2* transcription, we analyzed the *SOX2* promoter sequence and found one putative ZNF32-binding site (5′-gcattt-32) located at -1545 (Figure 4C). Based on the ZNF32-DNA binding site, a biotin-labeled *SOX2* probe was synthesized for EMSA. The *SOX2* probe was specifically bound by ZNF32 rather than by GST, and the binding complex was significantly attenuated by the addition of competing probes *in vitro* (Figure 4D). Furthermore, nuclear proteins extracted from the indicated BE(2)-C cell lines were used to perform EMSA and revealed that ZNF32 over-expression increased the formation of DNA-nucleoprotein complexes, whereas the interaction between proteins and probes was significantly decreased in ZNF32-silenced cells (Figure 4E). Next, a GFP antibody was used to immunoprecipitate GFP-tag protein-DNA complexes, with rabbit IgG antibody used as a negative control. Chromatin immunoprecipitation revealed that ZNF32 directly bound to the endogenous *SOX2* promoter (Figure 4F). Our data demonstrate that ZNF32 negatively regulates *SOX2* expression through direct binding to the *SOX2* promoter.

### Amino acids 1-169 are essential for ZNF32 nuclear localization

**Figure 5:**
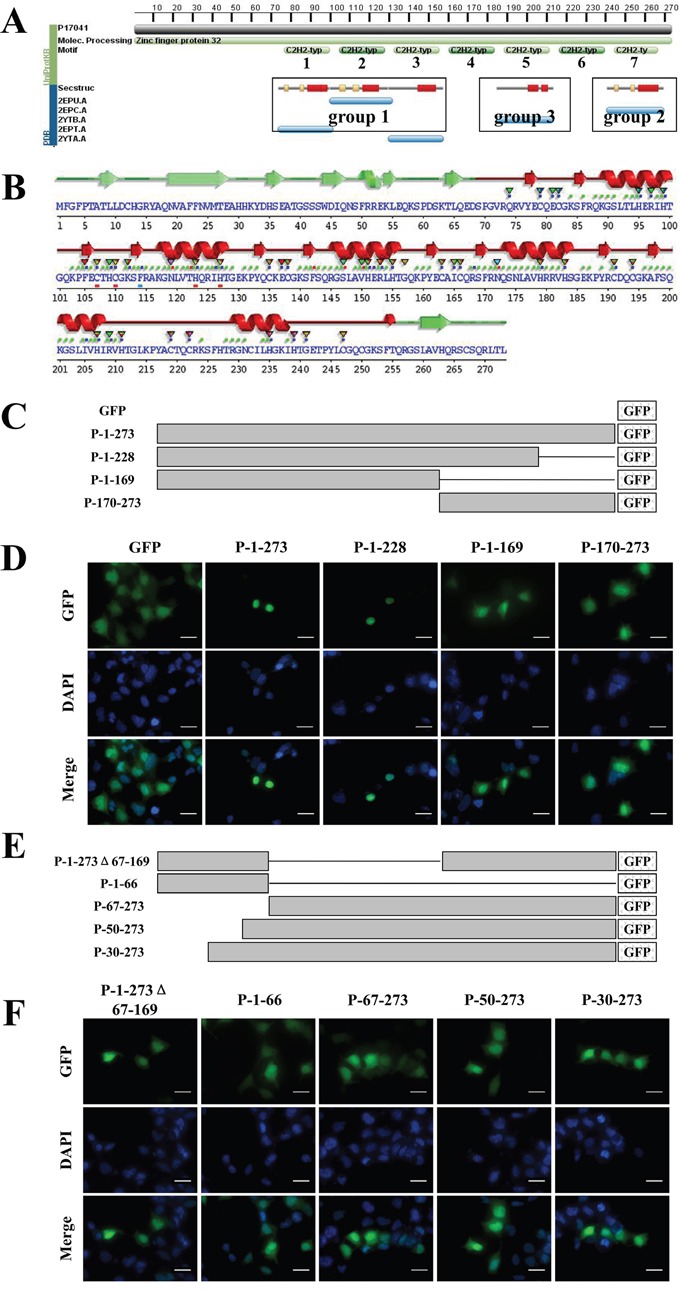
Bioinformatics prediction and the importance of Aa 1-169 of ZNF32 **A.** Screenshot from the PDB predicting the zinc-finger motifs and secondary/tertiary structural components. **B.** Screenshot from EMBL-EBI web server showing the α-helixes and β-sheets predicted in ZNF32. **C, E.** Schematic representation of the recombinant, GFP-tagged ZNF32 mutant proteins. Lines represent the deleted sequences in the proteins. **D, F.** The subcellular localization of ZNF32 mutant proteins. Recombinant proteins are shown in green (GFP), and cell nuclei are shown in blue (DAPI). Scale bar = 50 μm.

As described above, we demonstrated that ZNF32 inhibited *SOX2* transcription. The NLS of ZNF32 is critical for the nuclear translocation and function of ZNF32. Bioinformatics analyses from the PDB and the EMBL-EBI protein data bank identified the zinc-finger motifs, secondary and tertiary structure of ZNF32 (Figure 5A). Several α-helixes and β-sheets are distributed in ZNF32 (Figure 5B). We also analyzed the protein sequence of ZNF32 and found no consensus NLS sequences based on similarity to the classical SV40 and bipartite NLS sequences. Thus, to identify the NLS of ZNF32, a series of mutants were constructed according to the secondary and tertiary structure, as shown in Figure 5C. Fusion proteins P-1-273 (full-length ZNF32), P-1-228 (Aa 1-228) are nuclear localized, whereas fusion proteins P-1-169 and P-170-273 displayed increased accumulation of fluorescence in the cytoplasm, suggesting that the ZNF32 NLS may be located between amino acids 170-228 (Figure 5D). However, the NLS of ZNF32 alone was not sufficient for the nuclear location, as P-170-273 was distributed throughout the cell. To further examine whether sequence 1-169 containing group 1 (Aa 67-169) was essential for the nuclear location of ZNF32, we constructed several ZNF32 deletion mutants (Figures 5E). Protein P-1-273Δ67-169 (Aa 67-169 deleted) was present throughout the whole cell, indicating that group 1 is indispensable for ZNF32 nuclear localization. Moreover, fusion proteins P-1-66, P-67-273, P-50-273 and P-30-273 showed significant cytoplasmic localization (Figure 5F). Our data demonstrate that the entire Aa 1-169 region is essential for ZNF32 nuclear localization.

### Three NLSs control ZNF32 nuclear localization

**Figure 6:**
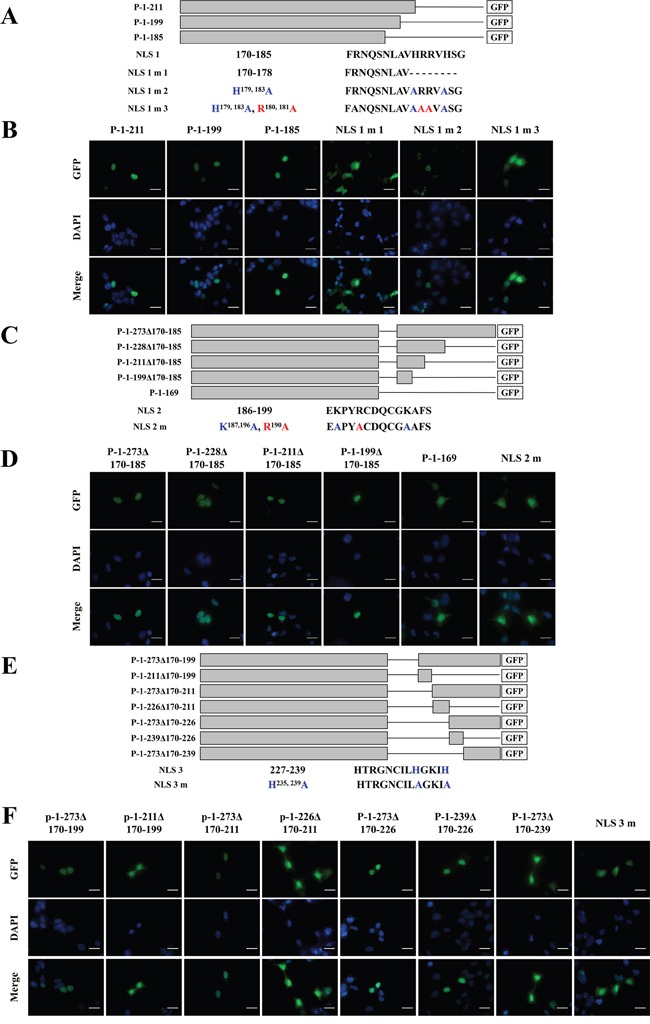
Identification of NLSs in ZNF32 and the localization of NLS mutants **A, B.** ZNF32 NLS 1 (Aa 170-185). (A) Schematic representation of recombinant, GFP-tagged ZNF32 mutant proteins. Lines represent the deleted sequences in the proteins. (B) The subcellular localization of ZNF32 and NLS 1 mutant proteins. Recombinant proteins are shown in green (GFP), and cell nuclei are shown in blue (DAPI). **C, D.** ZNF32 NLS 2 (Aa 186-199). The basic amino acids Lys and Arg were replaced with Ala in NLS 2 mutant. (C) Schematic representation of the recombinant, GFP-tagged ZNF32 mutants. Lines represent the deleted sequences in the proteins. (D) The subcellular localization of ZNF32 and NLS 2 mutant. Recombinant proteins are shown in green (GFP), and cell nuclei are shown in blue (DAPI). **E, F.** ZNF32 NLS 3 (Aa 227-239). (E) Schematic representation of the recombinant, GFP-tagged ZNF32 mutants. Lines represent the deleted sequences in the proteins. (F) The subcellular localization of ZNF32 and NLS 3 mutant. Recombinant proteins are shown in green (GFP), and cell nuclei are shown in blue (DAPI). Scale bar = 50 μm.

The above results showed that the NLSs of ZNF32 may be located between Aa 170-228 in the presence of entire Aa 1-169 region. Fusion proteins P-1-211, P-1-199 and P-1-185 were nuclear localized, suggesting the presence of one NLS at Aa 170-185 (named NLS 1). Numerous previous studies have shown that basic amino acids are important in NLSs [23, 24]. Hence, we constructed three mutations of NLS 1 (Figure 6A): NLS 1 m 1 (NLS 1 mutation 1, Aa 179-185 deleted in Aa 170-185) failed to undergo nuclear localization (Figure 6B); NLS 1 m 2 (H^179, 183^A) displayed punctate fluorescence similar to nuclear bodies that consist mostly of proteins and noncoding RNAs [25] (Figure 6B); and NLS 1 m 3 (H^179, 183^A and R^180, 181^A) was diffusely distributed throughout the whole cell (Figure 6B). The NLS 1 deletion mutations had no effect on the localization of the GFP fusion proteins P-1-273Δ170-185, P-1-228Δ170-185, P-1-211Δ170-185, and P-1-199Δ170-185, suggesting that Aa 186-199 may be another NLS (named NLS 2) (Figure 6C and 6D). Strong cytoplasmic fluorescence was observed in NLS 2 m (K^187, 196^A and R^190^A) (Figure 6C and 6D). Moreover, fusion proteins P-1-273Δ170-199, P-1-273Δ170-211, P-1-273Δ170-226 and P-1-239Δ170-226 were localized to the nuclear alone, whereas P-1-211Δ170-199, P-1-226Δ170-211 and P-1-273Δ170-239 showed cytoplasmic fluorescence, suggesting the presence of a third NLS is between Aa 227-239 (named NLS 3) but not Aa 240-273 (Figure 6E and 6F). NLS 3 m (H^235, 239^A) exhibited significant cytoplasmic fluorescence (Figure 6E and 6F). Taken together, our data confirmed the existence of three independent and paratactic NLSs in ZNF32, which together with Aa 1-169 help direct ZNF32 protein entry into the cell nucleus.

### The three paratactic NLSs of ZNF32 contribute to the regulation of *SOX2* expression

**Figure 7:**
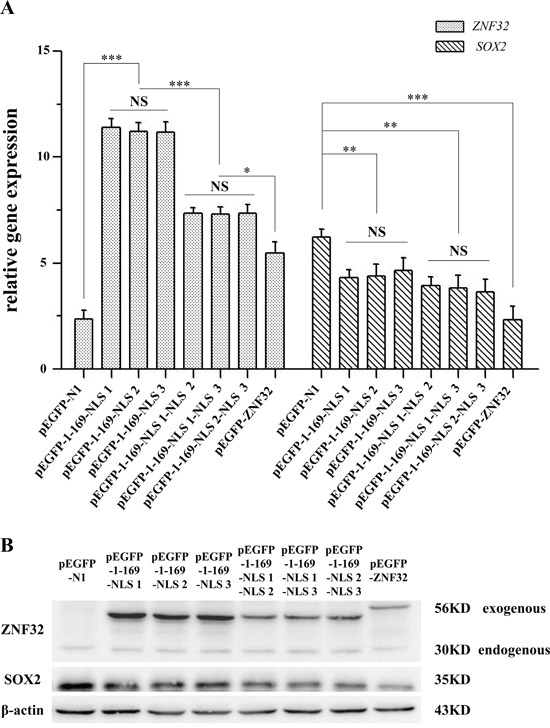
The NLSs of ZNF32 influence *SOX2* expression Several NLS-mutant *ZNF32* plasmids, pEGFP-1-169-NLS 1, pEGFP-1-169-NLS 2, pEGFP-1-169-NLS 3, pEGFP-1-169-NLS 1-NLS 2, pEGFP-1-169-NLS 1-NLS 3, pEGFP-1-169-NLS 2-NLS 3 and pEGFP-ZNF32, were transiently transfected into BE(2)-C cells. pEGFP-N1 was used as a negative control. The effects on *SOX2* expression were detected by Q-PCR **A.** and western blotting **B.** All of the quantitative values are presented as the means±S.D. *P< 0.05, **P< 0.01, ***P< 0.001. NS, not significant.

Next, to investigate whether the NLSs of ZNF32 were involved in the transcriptional regulation of *SOX2* by ZNF32, we constructed several NLS deletion mutations of ZNF32. Data from Q-PCR and western blotting showed that the pEGFP-N1, which displayed potent cytoplasmic localization and could not function as a TF, had no effect on *SOX2* expression. PEGFP-1-185 (pEGFP-1-169-NLS 1), pEGFP-1-199Δ170-185 (pEGFP-1-169-NLS 2) and pEGFP-1-239Δ170-226 (pEGFP-1-169-NLS 3) all entered the cell nucleus but showed less inhibition on *SOX2* expression compared with full-length pEGFP-ZNF32 (Figure 7A and 7B). Moreover, the suppression on *SOX2* expression by pEGFP-1-199 (pEGFP-1-169-NLS 1-NLS 2), pEGFP-1-239Δ186-226 (pEGFP-1-169-NLS 1-NLS 3) and pEGFP-1-239Δ170-185& Δ200-226 (pEGFP-1-169-NLS 2-NLS 3) was also weaker compared with pEGFP-ZNF32 (Figure 7A and 7B). These data suggest that the integrity of the ZNF32 protein structure is required for its transcriptional function.

## DISCUSSION

The externally located zebrafish lateral line is a powerful model for regeneration and is composed of rosette-shaped sensory organs called neuromasts [26]. Each neuromast contains a group of hair cells encased by a population of supporting cells [27]. Sensory hair cells, similar to the hair cells in terrestrial vertebrates, are constantly replaced and regenerated from surrounding non sensory cells [28]. Studies of the lateral line system provide important information regarding human hair cell regeneration after damage caused by age, drugs and so on. The lateral-line system comprises two major branches, an anterior Lateral Line (aLL) that extends on the head, and a posterior Lateral Line (pLL) that extends on the trunk and tail. The development of the pLL system was shown to involve the formation of a migrating primordium that deposits a prospective neuromast and migrates under the skin along the horizontal myoseptum to the tip of the tail [29]. In the present study, we found that *zfZNF32* is persistently expressed in the nervous system, especially in the lateral line system during zebrafish development. Knocking out *zfZNF32* enhanced the regenerative capacity of the lateral line system when hair cells were injured by neomycin. Moreover, downregulating *zfZNF32* increased *SOX2* expression, suggesting that *SOX2* was involved in the *zfZNF32*-mediated regeneration of the lateral line system. Indeed, suppression of *SOX2* significantly counteracted the elevated regenerative capacity of *zfZNF^−/−^*. Our findings reveal a novel and important mechanism by which *zfZNF32* prevents the regeneration of the nervous lateral line system via the negative regulation of *SOX2* expression. Recent studies have reported that *SOX2*, a neural progenitor marker expressed in all support cells, promotes the formation of newly born hair cells in neuromasts through progenitor cells, supporting cell proliferation, differentiation and direct transdifferentiation [30]. Furthermore, gene expression analysis following neomycin treatment reveal alterations the expression of several genes, including *SOX2* up-regulation [31]. In line with these studies, our results support the pivotal role of *SOX2* in the repair of the nervous system. Mammals have a very limited, if any, overall regenerative capacity compared with aquatic vertebrates. Thus, *ZNF32* can be used as a target gene for the treatment of deafness caused by hair cell damage in humans and other mammals.

The classical C2H2 zinc-finger domain is approximately 30 amino acids long and contains one amphipathic α-helix and two antiparallel β-strands in which two cysteines and two histidines coordinate a zinc ion to stabilize the structure for transcriptional regulation [32]. ZNFs bind DNA by inserting the α-helix into the major groove and use three or four exposed residues of the helix to make specific contacts with three or four DNA bases [33]. In the present study, we found that loss of *zfZNF32* augmented zebrafish lateral line system regeneration by negative regulation of *SOX2* expression. ZNF32 regulates *SOX2* expression by directly binding to a specific DNA binding site in the *SOX2* promoter. We also identified the consensus binding sequence of ZNF32 (5′-g(a/c/t)attt-32) by using CASTing. Moreover, we identified two additional ZNF32 binding sequences with lower frequency: 5′-gacttt-32 and 5′-gtcttt-32, which should be required further confirmation.

Nuclear localization signals are important for protein translocation from the cytoplasm to the nucleus. The prototypical NLS contains a cluster of basic amino acids located at any position in a nuclear protein and directs transport into the nucleus via the importin α/β heterodimer, Ran and Kapβs [34]. However, ZNF32 contains no classical nuclear localization signals. Aa 1-169 and another three separate NLSs are essential for ZNF32 transport to the nucleus. At least one NLS in the presence of Aa 1-169 can help the protein enter the cell nucleus. Many factors regulate protein localization, including amino acid modifications [35], stress [36] and protein mutations [37]. In the current study, ZNF32 protein mutants can change protein location-no nuclear location and nuclear body location. A series of ZNF32 mutants lacking NLSs failed to undergo nuclear transport and were therefore unable to functions as TFs, such as P-1-169. NLS 1 m 2, in which two His residues were mutated, localizes to nuclear bodies that contain various pre-mRNA editing regulators and are involved in exon recognition and alternative splicing. The subcellular location of ZNF32 NLS 1 m 2 implies that the NLS 1 m 2 protein interacts with proteins or RNAs in nuclear bodies rather than with DNA, as C2H2 zinc-finger proteins can also bind RNA, protein and/or lipid substrates [38]. When the localization of ZNF32 changed, its transcriptional regulation also declined. The suppression of *SOX2* expression by other ZNF32 protein mutants, such as pEGFP-185 and pEGFP-1-199, which contain only one or two NLSs, was weaker compared with full-length ZNF32. These data highlight the delicate and complex nature of transcriptional regulation. Protein mislocalization is related to many diseases such as Fused in Sarcoma-caused amyotrophic lateral sclerosis [39]. Given that protein mislocalization may lead to human diseases, our future studies will focus on ZNF32 protein mislocalization associated with diseases involved in the nervous system, wherein the NLSs of ZNF32 may provide targets for disease gene therapy in the future.

In our current study, *zfZNF32* was found to be expressed during zebrafish nervous system development, and the regenerative capacity of lateral line system was shown to be increased in *zfZNF^−/−^*. The nerve stem cell marker *SOX2* was negatively regulated by ZNF32 due to the binding of ZNF32 to a specific sequence in the *SOX2* promoter. ZNF32 is a nuclear protein that is critical for the transcriptional regulation of *SOX2* expression. Aa 1-169, NLS 1, NLS 2 and NLS 3 are vital for ZNF32 nuclear trafficking, and each NLS can influence ZNF32 transcriptional function. The clear regulatory mechanism of *SOX2* expression and the identification of NLSs in ZNF32 provide powerful foundations for future studies, including target regulation through protein NLSs and DNA binding site modifications.

## MATERIALS AND METHODS

### Zebrafish line and sequence retrieval

Wild-type embryos for all experiments were in the AB×Tubingen background. Embryos and zebrafish were bred, maintained and staged according to standard procedures [40]. The zebrafish *ZNF32* homologue was identified using HomoloGene at the National Center for Biotechnology Information. A homologue expressed sequence (*zfZNF32*) was used as a reference for the following experiments. The treatment and handing of zebrafish were performed under the general directives on the protection of animals used for scientific purposes and following operating procedures approved by the Sichuan Animal Care and Use Committee.

### CRISPR/Cas9-mediated zebrafish *zfZNF32* knock-out

CRISPR/Cas9 target sites in *zfZNF32* were designed online (ZIFIT Targeter: http://zifit.partners.org/zifit/Introduction.aspx), and the following sgRNA sequence was used: 5′-TAATACGACTCACTATAggtggtctttgagcttaccatggTTAGAGCTAGAAATAGCTGGTAA-32. The target site sequence was synthesized *in vitro* and inserted between the T7 promoter and the short guide RNA (gRNA) of pT7-gRNA. Cas9 mRNA and sgRNA were transcribed *in vitro* with T7 RNA polymerase. For CRISPR/Cas9 injection, Cas9 mRNA and sgRNA were mixed at a ratio of 5:3. The Cas9-*zfZNF3*2 mutations were identified, and the mutant zebrafish were obtained as previously stated [21]. Primers are listed in Table 1.

**Table 1:** Primers used in this study

Genes	Primers (up)	Primers (dw)
**pGEM-T-*ZfZNF32***	**up: ATGGCCTTTATTAAAGAGGAGAGTG**	**dw: TCAGATTTGATGGTTTCTGTCTTG**
**zebrafish *GAPDH* (Q-PCR)**	**up: GGAGTCTACTGGTGTCTTCACTACTATTG**	**dw: GAGGCATTGCTTACAACTGTGAGA**
***ZfZNF32* (Q-PCR)**	**up: GAGGAGAGTGAAGACCTGAAGATTG**	**dw: TCTACACCAGAGTTTTGACCATCC**
**zebrafish *SOX2* (Q-PCR)**	**up: CAGACCCTGATGAAGAAGGACAAG**	**dw: TGCATCTGCGCCGTGTTGT**
**homo *SOX2* (Q-PCR)**	**up: TGCAGTACAACTCCATGACCAG**	**dw: GGGAGGAAGAGGTAACCACAG**
**homo *ZNF32* (Q-PCR)**	**up: GAAGATATGCCCAGAATGTAGCG**	**dw: GGATTTTTGTTCCAGCTTCTCTCT**
**homo *GAPDH* (Q-PCR)**	**up: ACCACAGTCCATGCCATCAC**	**dw: TCCACCACCCTGTTGCTGTA**
**homo ChIP *SOX2***	**up: AAAGAAATGGCATCAGGTT**	**dw: CGCTACACGGAGTCAAAC**
***ZfZNF32* (for Cas9-*zfZNF32* zebrafish screening)**	**up: CCTTAGAGCTGACCTCGCTG**	**dw: CCACACTGAGGACACGTGAA**
**GFP-N1-ZNF32**	**up: GCGAATTCCGATGTTTGGATTTCC**	**dw: GAAGGTACCCAAAGGGTGAGCCTCT**
**GFP-N1-ZNF32 Aa 66**		**dw: GTGGTACCCCCTGTAGTGTCTTCGAAT**
**GFP-N1-ZNF32 Aa 169**		**dw: GGGGTACCACGCTTCTCTGACAAATAG**
**GFP-N1-ZNF32 Aa 226**		**dw: CCCGGTACCCTGGTGTGGAAACTCTT**
**GFP-N1-ZNF32 Aa 170**	**up: GCGAATTCGCTTCAGGAATCAGAGTAAC**	
**GFP-N1-ZNF32 Aa 178**		**dw: GGTACCTGAACAGCAAGGTTACTCTGATT**
**GFP-N1-ZNF32 Aa 185**		**dw: AGGGTACCACACCACTGTGAACTCTCC**
**GFP-N1-ZNF32 Aa 186**	**up: GCGAATTCGCGAGAAGCCCTATAGATG**	
**GFP-N1-ZNF32 Aa 199**		**dw: AGGGTACCTGACTGAAGGCTTTTCCACAC**
**GFP-N1-ZNF32 Aa 200**	**up: AGGAATTCGCCAGAAAGGAAGCTTAATTG**	
**GFP-N1-ZNF32 Aa 211**		**dw: CTTCGGTACCGTGTGGACTCTGATGTG**
**GFP-N1-ZNF32 Aa 212**	**up: CAGAATTCACACAGGCCTGAAGC**	
**GFP-N1-ZNF32 Aa 30**	**up: CGAATTCTCCACCACAAATATGACCAC**	
**GFP-N1-ZNF32 Aa 50**	**up: CGAATTCTCAGAAGAGAGAAGCTGGAAC**	
**GFP-N1-ZNF32 Aa 67**	**up: CAGAATTCCGGAAGATTCACCTGGAG**	
**GFP-N1-ZNF32 Aa 239**		**dw: AGGGTACCGTGTGGATTTTGCCATG**
**GFP-N1-1-185 H^179,183^A**		**dw: GGTACCGTACCACTCGCAACTCTCCTGGCAAC**
**GFP-N1-1-185 H^179,183^A, R^180,181^A**		**dw: GGTACCGTACCACTCGCAACTGCCGCGGCAAC**
**GFP-N1-1-199Δ170-185 K^187^A, R^190^A**		**dw: GGTACCCAATCACATGCATAGGGCGCCTC**
**GFP-N1-1-199Δ170-185 K^196^A**		**dw: GGTACCTGACTGAAGGCTGCTCC**
**GFP-N1-1-239Δ170-226 H^235, 239^A**		**dw: GGTACCGTAGCGATTTTGCCAGC**
**GFP-N1-ZNF32 Aa 66-200 overlap**	**up: ACACTACAGCAGAAAGGAAGCTTAATTG**	**dw: TTTCTGCTGTAGTGTCTTCGAATCTG**
**GFP-N1-ZNF32 Aa 169-212 overlap**	**up: GCTATTTGTCAGAGAAGCACAGGCCT**	**dw: AGGGCTTCAGGCCTGTGCTTCTCT**
**GFP-N1-ZNF32 Aa 169-227 overlap**	**up: CTATTTGTCAGAGAAGCCACACCAGG**	**dw: CAATTCCCCCTGGTGTGGCTTCTCTGAC**
**GFP-N1-ZNF32 Aa 169-240 overlap**	**up: GCTATTTGTCAGAGAAGCACAGGAGAGA**	**dw: ATAGGGTGTCTCTCCTGTGCTTCTCTG**
**GFP-N1-ZNF32 Aa 185-227 overlap**	**up: AGGAGAGTTCACAGTGGTCACACCAGGGGG**	**dw: TTCCCCCTGGTGTGACCACTGTGAACTCTC**
**GFP-N1-ZNF32 Aa 199-227 overlap**	**up: TGGAAAAGCCTTCAGTCACACCAGGGGGA**	**dw: TTCCCCCTGGTGTGACTGAAGGCTTTTCC**
**GST-ZNF32**	**up: CCAGGAATTCTATGTTTGGATTTC**	**dw: CTCGAGTCAAAGGGTGAGCCTCTGT**

Primers used for *zfZNF32* plasmid construction, Q-PCR gene amplification, ChIP assay and Cas9 mediated *zfZNF^−/−^* screening.

### Hair cell damage and regeneration

In order to study *zfZNF32* function in lateral line system regeneration, hair cells in zebrafish larvae were damaged via neomycin treatment. Z*fZNF^−/−^* at 4 dpf were treated with 400 μM neomycin (Amersco, China) for 1 hr, rinsed 4 times in fresh water, and then returned to the incubator in normal embryo medium at 28.5°C. Wild-type zebrafish were used as a control [41].

A *SOX2* morpholino (MO), 5′-GAA AGT CTA CCC CAC CAG CCG TAA A-32, was designed to block *SOX2* gene expression. A standard MO (5′-CCT CTT ACC TCA GTT ACA ATT TAT A-32) was used as a control [42]. All MOs were purchased from Gene Tools. MOs were microinjected to *zfZNF^−/−^* and WT embryos at the one-cell stage, then the hair cells of 4 dpf larvae were damaged as the above.

2-[4-(dimethylamino) styryl]-N-ethylpyridiniym iodide (DASPEI, Sigma) was used to stain hair cells in lateral line neuromasts. After 12 and 24 hrs, larvae were placed into 0.005% DASPEI in embryo medium for 15 min and then anesthetized in MS222 (10 μg/ml, 3-aminobenzoic acid ethyl ester, methanesulfonate salt; Sigma) for 5 min. DASPEI-labeled cells in neuromasts were analyzed using fluorescence microscopy (ECLIPSE TE2000-U, Nikon, Japan) [43]. At the same time, larvae were collected for Q-PCR.

### Whole-mount in situ-hybridization

The full coding sequence of *zfZNF32* was amplified from cDNA derived from 24 hpf embryos, and the primers are listed in Table 1. *ZfZNF32* cDNA was subcloned into pGEM-T-easy (Promega), yielding the plasmid T-*zfZNF32* for digoxigenin-labelled antisense riboprobe synthesis. *ZfZNF32*, *SOX2*, *nestin* and *olig2* probes were used with the DIG RNA labeling Mix and T7 RNA polymerase (Roche) according to the manufacturer's instructions. Primers are listed in Table 1. Whole-mount *in situ* hybridization (WISH) was carried out as previously described [44].

### RNA preparation and quantitative real-time PCR

Zebrafish embryos at different stages were snap-frozen in liquid nitrogen. Total RNA was extracted using RNAiso plus (TAKARA, Dalian, China) according to the manufacturer's instructions. cDNA was synthesized from RNA isolated at different stages, and Q-PCR was carried out using the SYBR Premix Ex Taq II kit (TAKARA, Dalian, China). Relative expression levels were determined using Gene expression Macro Version 1.1 software (BIO-RAD). Primers are listed in Table 1, and data are presented as the mean±S.D.

### Cell culture

Human embryonic kidney cells (HEK293) and human neuroblastoma BE(2)-C (from American Type Culture Collection) and BE(2)-C stably cell lines were each maintained in DMEM and EMEM medium (Gibco) containing 10% fetal bovine serum (Gibco), 100 U/mL penicillin, 100 mg/mL streptomycin and 3.7 g/L NaHCO_3_. Cells were maintained at 37°C in a humidified 95% air and 5% CO_2_ atmosphere.

The ZNF32 lentiviral expression vector was constructed by inserting ZNF32 cDNA into the LV6 lentiviral shuttle vector, yielding LV6-ZNF32. ShRNA targeting ZNF32 was cloned to the lentiviral vector - LV2pGLVU6/GFP + Puro, yielding lv-ZNF32. Recombinant lentiviral plasmids were transfected into BE(2)-C cells. Stable BE(2)-C cell lines were selected with puromycin. The packing and purification of recombinant lentiviral vectors was performed by Shanghai GenePharma Company. The shRNA sequences were as follows: shRNA-ZNF32, 5′-GAATGTAGCGTTCTTCAATGT-32; shRNA-NC, 5′-TTCTCCGAACGTGTCAGGT-32. The pSG5 vector (pSG5-Vec) was stored in our lab, and the pSG5-ZNF32 over-expression plasmid (pSG5-ZNF32) was constructed by inserting expanded ZNF32 cDNA fragments into pSG5-Vec.

### Cellular localization studies

Different lengths of DNA encoding amino acids, internal deletion mutations and point mutations of human *ZNF32* were amplified by PCR/overlapping PCR and cloned into the pEGFP-N1 vector. All constructs were confirmed by DNA sequencing. The primers used are listed in Table 1, and the enzymes used in this part were purchased from TAKARA. Recombinant plasmids were transiently transfected into HEK293 cells, and subcellular location assays were performed as described previously [45]. Transfected cells were analyzed by fluorescence microscopy (ECLIPSE TE2000-U, Nikon, Japan), and every experiment was repeated at least three times with similar results.

### Western blotting

Protein samples were gel fractionated and transferred to polyvinylidene difluoride (PVDF) membranes (Millipore). The primary antibodies used were rabbit anti-GST (1:2000, Santa Cruz Biotechnology) and mouse anti-ZNF32, which was from our previous study, mouse anti-β-actin (1:4000, Abcam, United Kindom), and rabbit anti-SOX2 (1:2000, CST, USA). Membranes were incubated with horseradish peroxidase (HRP) conjugated goat-anti-rabbit IgG (1:8000, Santa Cruz Biotechnology) or HRP-goat-anti-mouse IgG (1:8000, Abcam, United Kindom) secondary antibody for 1 hr at 37°C and then developed using Immobilon™ Western Chemiluminescent HRP Substrate (Millipore).

### Expression, purification and identification of the GST-ZNF32 fusion protein

Full-length *ZNF32* was cloned into the pGEX-5X-3 vector (GE Healthcare, United Kindom) and transformed into *E.coli* BL21(DE3) star competent cells. The fusion protein was expressed under optimum folding conditions [46], and protein samples were collected at each step for 12% sodium dodecyl sulfate-polyacrylamide gel electrophoresis (SDS-PAGE). Separated proteins were visualized with Coomassie brilliant blue R 250, and the expression level of the fusion protein was estimated by densitometric analysis with Quantity One 1D image analysis (Bio-Rad).

GST-ZNF32 fusion protein from the supernatant was applied to affinity chromatography with Glutathione Sepharose 4B (GE Healthcare, United Kindom) according to the manufacturer's instructions. Purified protein was digested with Factor Xa (GE Healthcare, United Kindom) for 16 hrs in 1 mM CaCl_2_, 100 mM NaCl, and 50 mM Tri-HCl (pH 8.0) at 22°C to remove the GST tag. Protein samples were used for Coomassie brilliant blue R 250 Staining and western blotting.

### Screening for ZNF32 DNA binding sites by CASTing

The nucleic acid sequence bound by ZNF32 was selected from a pool of random oligonucleotides via CASTing. A 55-base oligonucleotide was synthesized beginning with 5′-ACCACAGTCCATGCCATCAC-32, followed by a stretch of 15 random bases and ending with the sequence 5′-TCCACCACCCTGTTGCTGTA-32; the known sequences also served as priming sites for subsequent PCR. After synthesis of the complementary strand by PCR, the double-stranded oligomer was mixed with bead-GST-ZNF32 complexes, and the bound DNA was amplified by PCR. The products from the first CASTing were used for the next CASTing cycle [47]. Ten cycles of CASTing were performed, and the final PCR products that preferentially bound ZNF32 compared with GST were cloned into the pGEM-T easy vector. A total of 22 clones were sequenced.

### Electrophoretic mobility shift assay (EMSA)

In order to determine the relative affinity of ZNF32 for double-stranded DNA, the 5′-biotin-labeled oligonucleotide 5′-tttgtagtcaagtgcattttagccacaaagat-32, corresponding to the human *SOX2* promoter, was synthesized by Invitrogen. The GST-ZNF32 fusion protein was purified as described above. Nuclear proteins from BE(2)-C stable cells were prepared using the ProteoJET™ Cytoplasmic and Nuclear protein Extraction Kit (Thermo Scientific) according to the manufacturer's instructions. Approximately 10 μg of nuclear protein or 2 μg GST-ZNF32 fusion protein was incubated with 2 nM biotin-labelled double-stranded oligonucleotide probe in reaction buffer for 20 min at room temperature using the LightShift™ Chemiluminescent EMSA Kit (Pierce Biotechnology). Samples were subjected to 5% nondenaturing gel electrophoresis in 0.5×Tris-borate EDTA, transferred to a positively charged nylon membrane (Millipore), and the membrane was developed using the Chemiluminescent Nucleic Acid Detection Module (Thermo Scientific) according to the manufacturer's instructions. For competition assays, a 200-fold excess of unlabeled probe was included to the binding reactions.

### Chromatin immunoprecipitation (ChIP)

ChIP was performed using the EZ-Magna-ChIP TM G One-Day Chromatin Immunoprecipitation Kit (17-409 Millipore) according to the manufacturer's instructions. BE(2)-C cells transfected with pEGFP-N1/pEGFP-ZNF32 were cross-linked with 1% formaldehyde for 15 min at room temperature. Formaldehyde was quenched with 125 mM Glycine for 5 min, and the cells were then collected and washed. Cells and cell nuclei were lysed sequentially. The extract was sonicated and incubated overnight with anti-GFP antibody (ab183734, Abcam, United Kindom) and anti-IgG (ab171870, Abcam, United Kindom) at 4°C. The bound DNA was analyzed using PCR. The primers are listed in Table 1.

## SUPPLEMENTARY FIGURES



## Abbreviations

Cas: CRISPR-associated systems
CASTing: cyclic amplification and select of targets
ChIP: Chromatin immunoprecipitation
CRISPR: Clustered regularly interspaced short palindromic repeats
dpf: day post-fertilization
EMSA: Electrophoretic mobility shift assays
GFP: Green fluorescent protein
GST: glutathione S-transferase
hpf: hour post-fertilization
NLS: Nuclear Localization Signal
Olig2: oligodendrocyte transcription factor 2
shRNA: short hairpin RNA
SOX2: SRY-related HMG-box gene 2
WT: wide type
zfZNF32: zebrafish zinc finger protein 32
zfZNF-/-: zfZNF32 knock-out zebrafish
ZNF32: homo zinc finger protein 32
