# Correction: Loss of *ZNF32* augments the regeneration of nervous lateral line system through negative regulation of *SOX2 *transcription

**DOI:** 10.18632/oncotarget.27337

**Published:** 2019-12-24

**Authors:** Yuyan Wei, Kai Li, Shaohua Yao, Junping Gao, Jun Li, Yanna Shang, Jie Zhang, Le Zhang, Yanyan Li, Xianming Mo, Wentong Meng, Rong Xiang, Jiankun Hu, Ping Lin, Yuquan Wei

**Affiliations:** ^1^ Division of Experimental Oncology, State Key Laboratory of Biotherapy, West China Hospital, Sichuan University, and Collaborative Innovation Center for Biotherapy, Chengdu, P.R. China; ^2^ Division of Cancer Biotherapy, State Key Laboratory of Biotherapy, West China Hospital, Sichuan University, and Collaborative Innovation Center for Biotherapy, Chengdu, P.R. China; ^3^ Laboratory of Stem Cell Biology, State Key Laboratory of Biotherapy, West China Hospital, Sichuan University, and Collaborative Innovation Center for Biotherapy, Chengdu, P.R. China; ^4^ Department of Gastrointestinal Surgery and Laboratory of Gastric Cancer, State Key Laboratory of Biotherapy, West China Hospital, Sichuan University, and Collaborative Innovation Center for Biotherapy, Chengdu, P.R. China; ^5^ Department of Clinical Medicine, School of Medicine, Nankai University and Collaborative Innovation Center for Biotherapy, Tianjin, P.R. China

**This article has been corrected:** In Figure 6F, the images of P-1-169 group from Figure 5D were accidentally duplicated. The corrected Figure 6 is shown below. The authors declare that these corrections do not change the results or conclusions of this paper.

Original article: Oncotarget. 2016; 7:70420–70436. 70420-70436. https://doi.org/10.18632/oncotarget.11895

**Figure 6:**
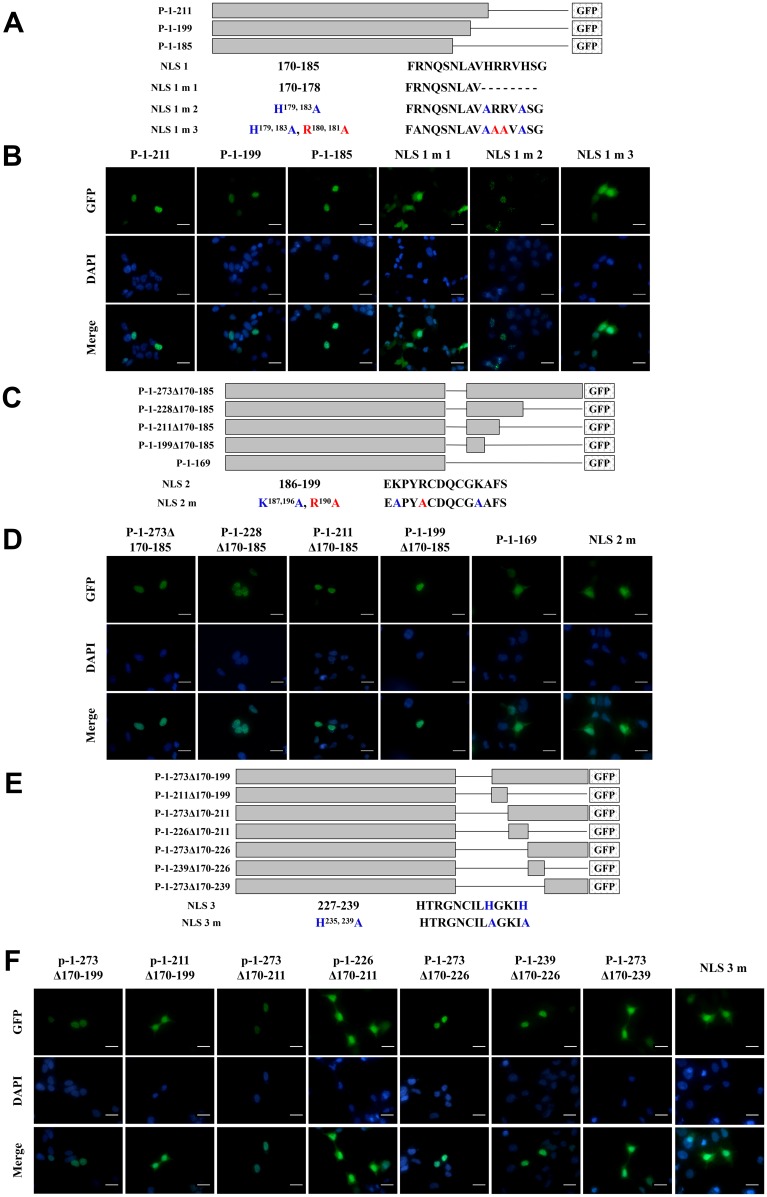
Identification of NLSs in ZNF32 and the localization of NLS mutants.

(**A**, **B**) ZNF32 NLS 1 (Aa 170-185). (A) Schematic representation of recombinant, GFP-tagged ZNF32 mutant proteins. Lines represent the deleted sequences in the proteins. (B) The subcellular localization of ZNF32 and NLS 1 mutant proteins. Recombinant proteins are shown in green (GFP), and cell nuclei are shown in blue (DAPI). (**C**, **D**) ZNF32 NLS 2 (Aa 186-199). The basic amino acids Lys and Arg were replaced with Ala in NLS 2 mutant. (C) Schematic representation of the recombinant, GFP-tagged ZNF32 mutants. Lines represent the deleted sequences in the proteins. (D) The subcellular localization of ZNF32 and NLS 2 mutant. Recombinant proteins are shown in green (GFP), and cell nuclei are shown in blue (DAPI). (**E**, **F**) ZNF32 NLS 3 (Aa 227-239). (E) Schematic representation of the recombinant, GFP-tagged ZNF32 mutants. Lines represent the deleted sequences in the proteins. (F) The subcellular localization of ZNF32 and NLS 3 mutant. Recombinant proteins are shown in green (GFP), and cell nuclei are shown in blue (DAPI). Scale bar = 50 μm.

