# Supplementary material for: Loss of ZNF32 augments the regeneration of nervous lateral line system through negative regulation of SOX2 transcription
Source: Oncotarget. 2016 Sep 8;7(43):70420–36. doi: 10.18632/oncotarget.11895 (PMC5342562; doi:10.18632/oncotarget.11895)
Supplement: Supplementary file 1 [file oncotarget-07-70420-s001.pdf]

Loss of *ZNF32* augments the regeneration of nervous lateral line system through negative regulation of *SOX2* transcription

SUPPLEMENTARY FIGURES

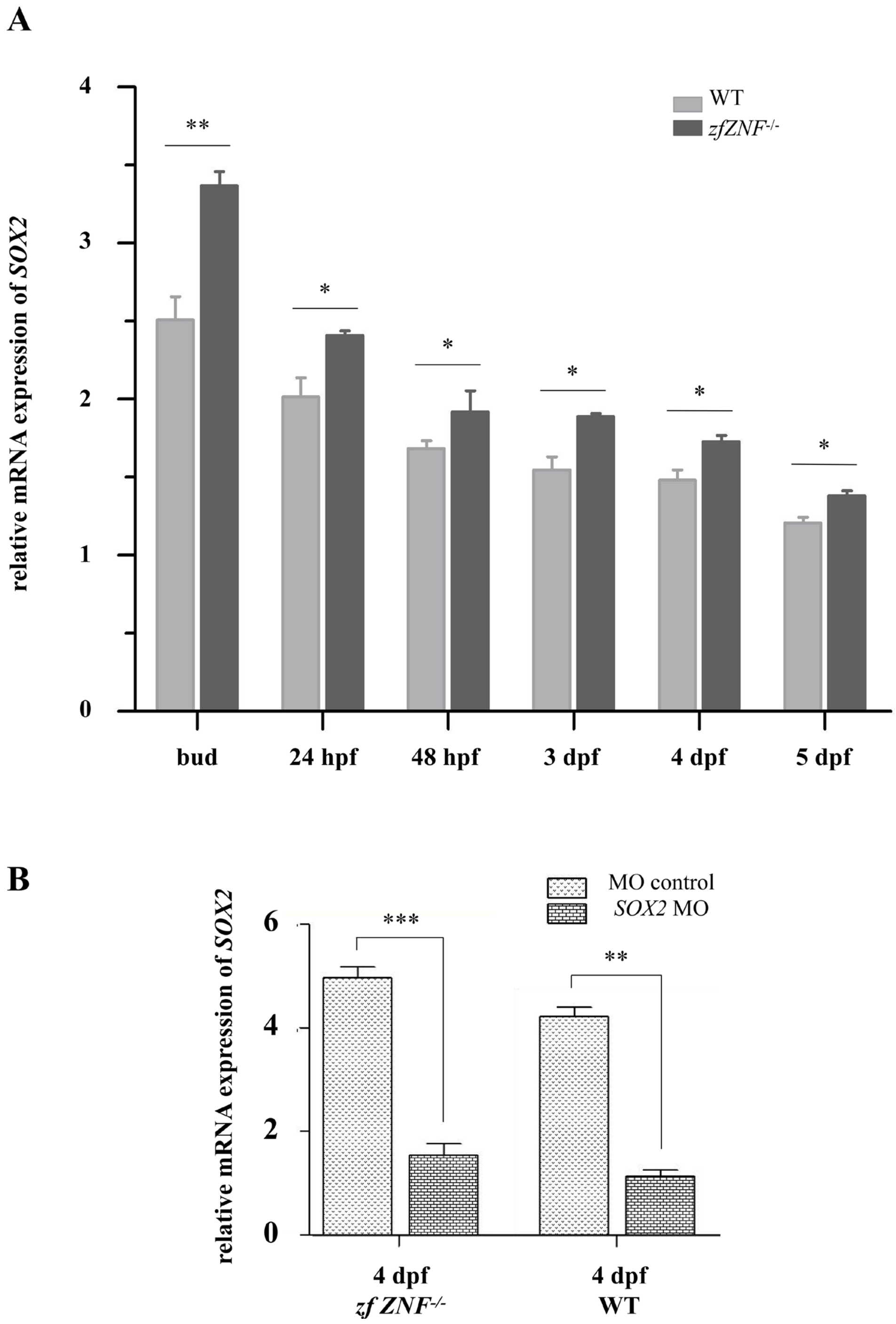

Supplementary Figure S1: Relative *SOX2* expression in WT and *zfZNF*$^{-/-}$ at different stage and with MOs microinjected. **A.** Q-PCR showing relative *SOX2* expression in WT and *zfZNF*$^{-/-}$ embryos at bud, 24 hpf, 48 hpf, 3 dpf, 4 dpf and 5 dpf stages. **B.** Q-PCR showing relative *SOX2* expression in 4 dpf WT and *zfZNF*$^{-/-}$ with *SOX2* MO or control MO microinjected. All of the quantitative values are presented as the means±S.D. *P< 0.05, **P< 0.01, ***P< 0.001.

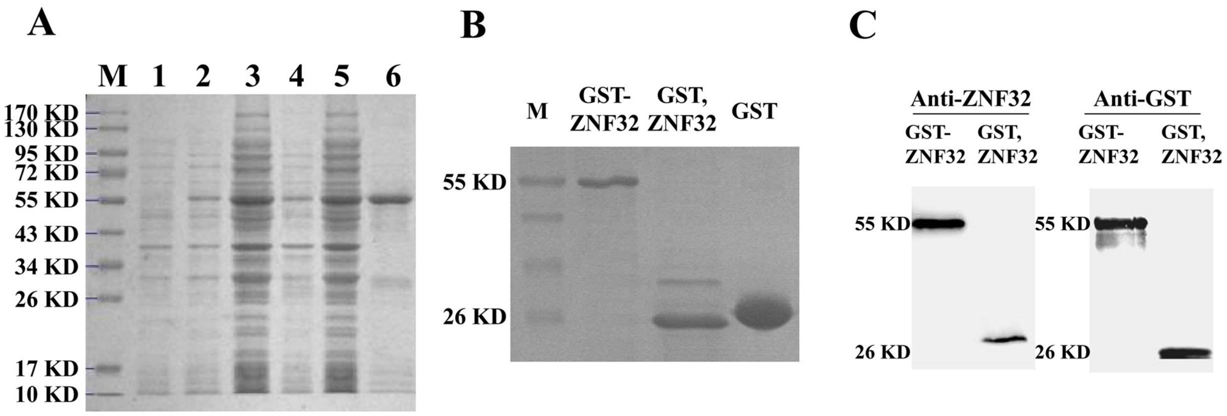

Supplementary Figure S2: GST-ZNF32 protein expression, purification and identification. A. SDS-PAGE showing GST-ZNF32 protein expression following overnight induction with 1 mM IPTG at 18°C. Lane 1, protein marker. Lane 2, whole *E. coli* lysate before IPTG induction. Lane 3, whole-cell lysate after overnight IPTG induction. Lane 4, insoluble material. Lane 5, cell lysate after the first round of affinity purification. Lane 6, affinity-purified GST-ZNF32 fusion protein. The fusion protein and its Factor Xa digestion products were identified by SDS-PAGE **B.** and western blotting **C.**
